# Significant structural change in human c-Myc promoter G-quadruplex upon peptide binding in potassium†

**DOI:** 10.1039/d2ra00535b

**Published:** 2022-03-08

**Authors:** Nikita Kundu, Taniya Sharma, Sarvpreet Kaur, Mamta Singh, Vinit Kumar, Uttam Sharma, Aklank Jain, Jadala Shankaraswamy, Daisuke Miyoshi, Sarika Saxena

**Affiliations:** Amity Institute of Biotechnology, Amity University Uttar Pradesh, Structural Biology Lab Sector-125, Expressway Highway Noida 201313 India ssaxena1@amity.edu sarikaigib@yahoo.co.in +91-120-4735600; Amity Institute of Molecular Medicine and Stem Cell Research, Amity University Uttar Pradesh Noida 201313 India; Department of Animal Sciences, Central University of Punjab Bathinda India; Department of Fruit Science, College of Horticulture, Mojerla, Sri Konda Laxman Telangana State Horticultural University 509382 Telangana India; Faculty of Frontiers of Innovative Research in Science and Technology (FIRST), Konan University 7-1-20 Minatojima-minamimachi, Chuo-ku, Kobe Hyogo 650-0047 Japan

## Abstract

We selected the G-quadruplex motif located in the nuclease-hypersensitive elements (NHE) III1 region of the c-Myc promoter and for the first time performed its interaction studies with a designed peptide (QW10). Our CD results showed that the peptide bound to the c-Myc G-quadruplex and induced a significant blue shift in the positive peak of 20 nm in KCl alone or with 40wt% PEG200 or 20wt% PEG8000 in comparison to NaCl. Our Native Gel results confirmed that peptide binding destabilized the duplex and stabilized the unimolecular G-quadruplex and not binding to i-motif. UV thermal results confirmed destabilization of bimolecular structure and stabilization of unimolecular G-quadruplex. QW10 showed preferential binding towards c-MYC promoter G4 with binding constant (*K*_b_) values of the order of 0.05 ± 0.2 μM, 0.12 ± 0.1 μM and 0.05 ± 0.3 μM for complexes in K^+^ alone or 40wt% PEG 200 or 20wt% PEG 8000 respectively. QW10 showed preferential cytotoxicity with IC_50_ values of 11.10 μM and 6.44 μM after 72 and 96 hours' incubation on Human Breast Carcinoma MDA-MB 231 cells and was found to be non-toxic with Human Embryonic Kidney (HEK-1) cells. Interestingly, we observed reduction of c-Myc gene expression by 2.5 fold due to QW10 binding and stabilizing c-MYC G4. Our study for the first time provides an expanded overview of significant structural change in human c-Myc promoter G-quadruplex upon peptide binding in potassium.

## Abstract 2

We selected the G-quadruplex motif located in the nuclease-hypersensitive elements (NHE) III1 region of the c-Myc promoter and for the first time performed its interaction studies with a designed peptide (QW10).

## Graphical abstract

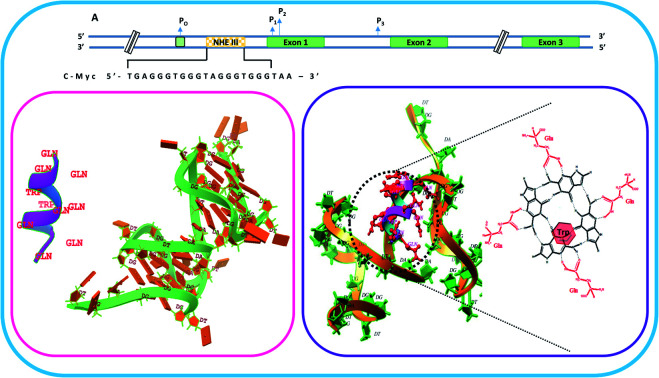

## 1 Introduction

G-quadruplexes are one of the higher-order DNA structures which have been identified for their functional importance in the regions of the genome like human telomeres and oncogene promoters. In the human genome, about 376 000 putative motifs are present, which tend to form a G-quadruplex structure.^1,2^ Depending upon their location, G-quadruplex motifs act as gene regulators *via* regulating various essential biological processes like telomeric maintenance and control the transcription and translation of various protooncogene oncogene's promoter (c-Myc, c-kit, k-ras, and bcl-2) regions.^3–10^ c-Myc is an important regulator for the processes of normal cellular growth regulation and differentiation, and its dysregulation is one of the hallmarks of many cancers. The abnormal over-expression of c-Myc has been reported in human cancers such as breast, colon, and cervix carcinomas, as well as small-cell lung cancer, glioblastomas, osteosarcomas, and myeloid leukemia.^11^ The promoter region located 142–115 bp upstream from the P1 promoter of the c-Myc gene is composed of seven (NHEs), of which, NHE III1 controls 80–90% transcription of c-Myc gene and is responsible for mediating multiple pathways important in tumor cell survival. The (NHE) III1 region of the c-Myc promoter can fold up into the G-quadruplex structure,^12,13^ and compounds that stabilize the G-quadruplex can repress c-Myc gene expression.^13–16^ This could be due to the polymerase pausing at the site of G4 formation or resulted in altered protein expression and also can activate DNA damage responses, thereby potentiating their anticancer effects. Therefore, small molecule that binds and stabilizes G4 DNAs leading to be considered as an alternative and a promising strategy to effectively inhibit the growth of cancer cells and overcome the problems of drug resistance.^17^ Over the past decades, several derivatives of quindolines have shown effective to stabilize the c-Myc G-quadruplex and down-regulate the expression of c-Myc in cancer cells.^19,20^ In recent years, a number of metal complexes like ruthenium(ii) have been reported to effectively bind to, induce and stabilize c-Myc G-quadruplex DNA.^21,22^ Zhang *et al.* have been synthesized and characterized the complex [Ru(phen)2(*p*-tFMPIP)](ClO_4_)_2_ {*p*-tFMPIP = 2-(4-(trifluoromethyphenyl)-1*H*-imidazo[4,5*f*][1,10]phenanthroline)} to stabilize the c-Myc G-quadruplex DNA and showed the growth inhibition in MDA-MB-231 cells through apoptosis pathway.^18^ Li *et al.* reported a series of aryl alkyne modified complex [Ru(phen)_2_(*p*-tFMPIP)](ClO_4_)_2_ {*p*-tFMPIP = 2-(4-(trifluoromethyphenyl)-1*H*-imidazo[4,5*f*][1,10]phenanthroline)} as a groove binder of c-Myc G-quadruplex DNA which can also act as a potential luminescent switch-on probe through selectively recognizing and promoting self-assembly of c-Myc G-quadruplex DNA.^21^ Wu *et al.* demonstrated that a series of arene ruthenium(ii) complexes [(η_6_-C_6_H_6_)Ru(*p*-XPIP)Cl]Cl (X = H; F; Cl; Br; and I) {PIP = 2-phenylimidazole[4,5*f*][1,10]phenanthroline)} which were binding and stabilizing c-Myc G-quadruplex DNA and exhibit excellent inhibitory activity against MDA-MB-231 breast cancer cells.^23^ Recently, bioactive compound L755507 was reported as novel inhibitor blocks c-Myc–MAX heterodimerization and induces apoptosis in cancer cells.^24^ Despite the progress in the discovery of new G-quadruplex-interactive ligands, important questions remain regarding the ligand-binding specificity in the c-Myc G-quadruplex. To date, no small molecule inhibitors that directly target the c-Myc interaction have progressed to clinical trials except few report.^25^ This is due to less target selectivity, rapid metabolism and low potency.

We addressed this issue by using a designed peptide, QW10 (QQWQQQQWQQ), which may be possible to bind and stabilize c-Myc promoter G4 with high selectivity in cation specific manner. In the peptide, glutamine (Q) residues possibly may bind to the hydrogen bonding donor and acceptor sites with guanine bases followed by the intercalation of tryptophan residues. Based on the molecular design, it is possible to consider that binding modes of QW10 as hydrogen bonding and stacking interaction. In this study, we have used 22-mer studied c-Myc promoter G-quadruplex sequence and performed its interaction with designed peptide QW10. Biophysical studies such as circular dichroism, fluorescence spectroscopy, UV-thermal melting and biochemical studies Native Gel Electrophoresis were employed to investigate the binding mode of QW10 with c-Myc promoter G-quadruplex structure. Our results clearly showed that the peptide binds with c-Myc promoter G4 in cation specific manner and showed structural distinctiveness, binding with Hoogsteen bonded G-quadruplex, destabilizing the Watson–Crick hydrogen bonded DNA duplex and not binding with i-motif structure. This unique design of peptide makes the peptide structure specific and cation selective G4 targeting ligand which may open the new ways for selecting the peptide as a unique drug molecule in future for cancer patients. Furthermore, *in vitro* studies were also employed to understand the cytotoxic effects of QW10 and its subcellular localization showing its potential to down-regulate c-Myc expression in human breast carcinoma cells and found to be non-toxic to human embryonic kidney cells. Hence, the present binding studies for the first time reveal important hints on the relationship between the structure and the selective binding of the peptide as next-generation G-4 targeting natural ligands for potential therapeutic treatment of cancer.

## 2 Materials and methods

### 2.1 DNA and peptide

PAGE purified grade DNA oligonucleotide and HPLC purified peptide [QQWQQQQWQQ] was purchased from Helix Biosciences. The concentration of the peptide was determined by measuring the absorbance of Trp at the C-terminal at 280 nm at 25 °C. Single-strand concentrations of DNA oligonucleotides were determined by measuring the absorbance at 260 nm at a high temperature using a Shimadzu 1800 Spectrophotometer (Shimadzu, Tokyo, Japan) connected to a thermoprogrammer. Single-strand extinction coefficients were calculated from mononucleotide and dinucleotide data using the nearest-neighbour approximation.^26,27^

### 2.2 Circular dichroism spectroscopy

CD spectra were carried out on JASCO-715 spectropolarimeter using a quartz cuvette of 1 cm path length. All the spectra were recorded in the range of 200–350 nm wavelengths at a scanning rate of 100 nm min^−1^. Before measurement, the samples were heated to 95 °C in water bath and slowly cooled till water attains room temperature and incubated at 4 °C overnight to avoid any non-equilibrium structures. Average scans of the DNA samples were subtracted from the buffer scan and data was normalized as a function of DNA strand concentration and path length of the cuvette.

### 2.3 Thermal melting analysis

UV absorbance of different samples were recorded with a Shimadzu 1800 spectrophotometer (Shimadzu, Tokyo, Japan) equipped with a temperature controller. Melting curves of DNA structures were obtained by measuring the UV absorbance at 260 nm or 295 nm in buffer pH 7.0 containing 0.5 mM EDTA, 100 mM NaCl, or 100 mM KCl, with and without 40 wt% PEG 400 or 20 wt% PEG 8000 in the presence or absence of QW10 peptide at DNA : peptide ratio (1 : 0), (1 : 1), (1 : 2), (1 : 5) and (1 : 10). The *T*_m_ values for 4 μM DNA structures were obtained from the UV melting curves as described previously (ref). The heating rates were 0.5 °C min^−1^. Before measurement, the samples were heated to 95 °C in water bath and slowly cooled till water attains room temperature and incubated at 4 °C overnight to avoid any non-equilibrium structures. Experiment has been repeated in triplicates to reproduce the data.

### 2.4 Native gel electrophoresis

For doing native gel experiment, 15% (w/v) polyacrylamide gel was used. Here in PAGE experiment, samples were prepared in 30 mM sodium cacodylate buffer (pH 7.4) containing 0.5 mM EDTA, 100 mM NaCl or 100 mM KCl, with and without 40 wt% PEG 200. The samples were heated to 95 °C in water bath and slowly cooled till water attains room temperature and incubated at 4 °C overnight. The running buffer TBE (pH 7.4) also contains the same concentration of salt and EDTA in gel as contained in oligonucleotide sample. Experiment was performed in cold room at constant 50 V. A 1 : 1 mixture of glycerol and orange-G was used for tracking the movement of DNA oligonucleotides in the gel. Finally, gel was stained using silver staining and imaged using Gel-Doc (Biorad, Gurgaon, Haryana, India).

### 2.5 Fluorescence measurements

Fluorescence experiments were performed by utilizing a JASCO FP 8300 spectrofluorometer (JASCO, Tokyo, Japan). Experiments were carried out at 25 °C in 1 cm path-length quartz cuvette. Peptide 4 μM was taken in 30 mM buffer of pH 7.0 containing 100 mM NaCl or 100 mM KCl, 0.5 mM EDTA with and without 40 wt% PEG 200 titrated with equimolar concentration of c-Myc G-quadruplex. The temperature of the cell holder was regulated by a JASCO ETC-273T temperature controller. Samples were prepared by same procedure. Excitation and emission slit width were 5 nm each and the samples were excited at 275 nm and the emission was recorded in a range of 300 nm to 500 nm. Experiment has been repeated in triplicates to reproduce the data. The fluorescence intensity of QW10 was plotted at 342 nm, 347 nm and 343 nm against DNA concentration in the presence of K^+^ alone or with 40 wt% PEG 200 or 20 wt% PEG 8000.

### 2.6 *In vitro* cytotoxic assay of peptide QW10

#### 2.6.1 Cell culture

MDA-MB-231 (Epithelial) cells and human embryonic kidney (HEK-1) cells were procured from National Centre for Cell Sciences (NCCS), Pune, India, and grown in Roswell Park Memorial Institute Medium, RPMI (Sigma Aldrich, USA). Epithelial Human breast cancer cell line MDA-MB-231 and HEK-1 were cell culture grown in 10% FBS (Gibco) and 1% HEPES (Gibco) supplemented RPMI medium (Sigma) with 1% penicillin/streptoMycin (Gibco) in 5% CO_2_, humidified incubator at 37 °C. QW10 peptide-40 μM was dissolved in RPMI medium completely. The cells were maintained at 37 °C in a 5% CO_2_ humidified incubator using standard cell culture procedures. After 24 hours, the growth medium was removed and freshly prepared QW10 peptide stock was eleven times serially diluted by two-fold dilution (40 μM, 20 μM, 10 μM, 5 μM, 2.5 μM, 1.25 μM, 625 nM, 312 nM, 150 nM, 78 nM, 39 nM) and each concentration of 100 μL was added in triplicates to the respective wells and incubated at standard conditions. Non-treated control cells were also maintained in the same conditions to compare the growth inhibition. The content in all respective wells including tests and control was decanted after 72 and 96 hours of treatment and 20 μL of reconstituted MTT (Sigma) was added. After 2 hours of dark incubation in a 5% CO2 humidified incubator, the supernatant was removed and 100 μL of MTT solubilisation solution was added and kept in a shaking incubator at 37 °C to solubilize formazan crystals. The absorbance was recorded at 570 nm using a microplate reader. The IC_50_ value was analyzed using GraphPad prism 8.

### 2.7 Gene expression of QW10 peptide using RT-PCR

We applied this qPCR to analyse the response of MDA-MB-231 cells towards QW10 peptides. QW10 peptide (100 μM) was dissolved in double distilled water to prepare its aqueous solution. MDA-MB-231 cells were grown at an exponential growth phase and subjected to QW10 treatment at two different concentrations (5 μM and 10 μM) for 24 hours. Non-treated cells were maintained at the same conditions to compare the gene expression profile. For RNA isolation, the sample content was removed and the cells were washed with phosphate buffer saline (PBS, Gibco) followed by triazole washing. We have added chloroform and centrifuged at 12 000 rcf at 4 °C for 15 min. The supernatant was aliquoted and added isopropanol and centrifuged at 12 000 rcf at 4 °C for 15 min. Supernatant was discarded, 70% ethanol was added and centrifuged at 7500 rcf at 4 °C for 5 min. Ethanol was removed and nuclease-free water was added to resuspend the RNA. The RNA concentration was quantified with Nanodrop (Multiskan) and RNA integrity was evaluated through 1.3% agarose gel electrophoresis. For cDNA isolation, for each sample, a master mix (5× buffer, 10 μM dNTPs, RNase inhibitor, revert aid, and random primers) was prepared. To prepare cDNA, each PCR well contains 1000 ng RNA, master mix, and nuclease-free water. A short spin was used followed by a PCR run for 1 hour 10 minutes.

qPCR was performed with applied biosystems step one plus real-time PCR system using a final volume reaction of 10 μL containing forward and reverse primer, 1× SYBR Green master mix, and cDNA. The following thermal profile was applied: 1 cycle at 95 °C for 10 min, 40 cycles at 95 °C for 10 s, 64 °C for 30 s and 72 °C for 15 s. Melting curve analysis was performed at ramping from 60 °C to 90 °C and rising by 0.5 °C every 2 s. Gene expression variations were analyzed in terms of fold induction concerning the untreated control cells by 2^−ΔΔCT^ method. All experiments were conducted in triplicates.

#### 2.7.1 Designing of peptide

**Fig. 1:**
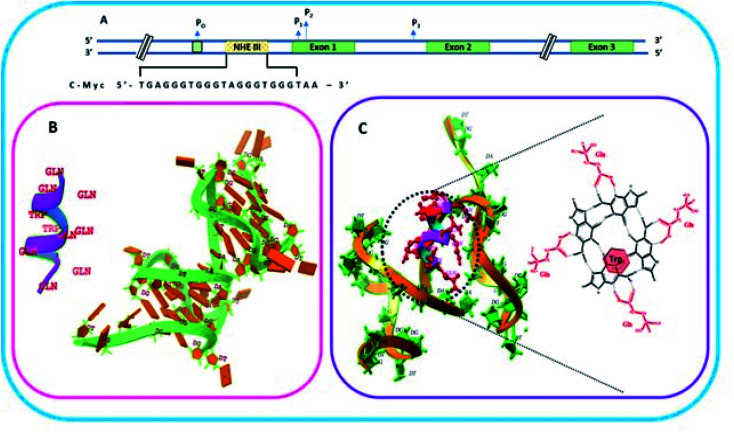
Schematic representation of c-Myc gene promoter structure. (A) Sequence that forms G quadruplex in the Nuclease Hypersensitivity Element III (NHE III1) located upstream of the Pl promoter. (B) Helical structure and molecular model of the peptide QW10 and c-MYC G4 (PDB I.D.: 6A1_41). (C) Molecular clocking of QW10 *versus*6AU4 with representation of binding of glutamine with G base in G-quartet plane followed hy the interaction of tryptophan.

QW10 (QQWQQQQWQQ) is a designed peptide which is glutamine rich containing intermittent tryptophan residues. This peptide is designed to make it structure selective as glutamine contains carbonyl group and amino group which can bind to the hydrogen bonding sites of G base, hence the idea is to allow the binding of glutamine with each G so when all four Gs within G quartet plane will get saturated due to glutamine binding then tryptophan may intercalate within G-quartet planes. Hence, this arrangement of the recognition of glutamine with G base by hydrogen bonding makes the peptide structure selective and intercalation of tryptophan residues may further affect the geometry of the structure. We have systematically shown the arrangement of G-quadruplex forming motifs located in the NHE1 site in the promoter region of c-Myc gene (Fig. 1a). Fig. 1b shows the stages of binding of QW10 with c-Myc G4 motif, initially binding with G-dimer, destabilizing it and promoting the folding into unimolecular G-quadruplex. Fig. 1c shows the binding mode of glutamine with G base within G-quartet plane followed by the intercalation of tryptophan.

#### 2.7.2 Structural changes in human c-Myc promoter G4 in the presence of Na^+^ or K^+^ with and without peptide under dilute conditions

**Fig. 2:**
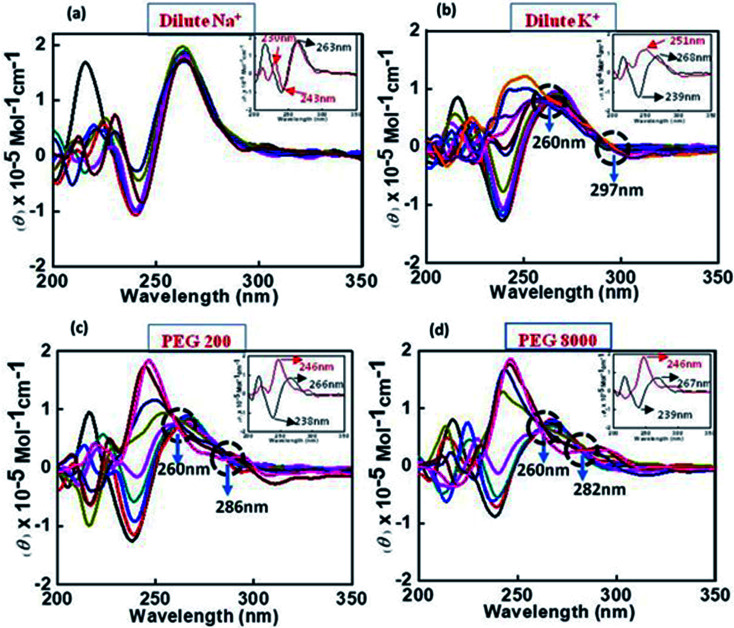
CD spectra of 4μM c-Myc G4 in buffer containing 0.5 mM EDTA, 100 mM NaC1 (a), 100 mM KCl (b), 100 mM KCl and 40 wt% PEG 200 (c), 100 mM KCl and 20 wt% PEG 8000 without any additive (black line) and titrated with an increase in concentration of QW10 peptide.

CD spectroscopy was employed to investigate the changes in the conformation of human c-Myc promoter G4 (c-Myc 5′-TGAGGGTGGGTAGGGTGGGTAA-3′) upon peptide binding. The structure of each DNA strand was recorded in the presence of 30 mM sodium cacodylate buffer pH (7.0) and 100 mM Na^+^ or 100 mM K^+^ in presence and absence of peptide (Fig. 2a). CD spectrum in 100 mM Na^+^ is characterized by a positive peak at 263 nm and negative peak at 243 nm, typically observed for parallel G-quadruplex in the presence of Na^+^.^28–30^ Next, human c-Myc promoter G4 was titrated with increasing concentration of QW10 to determine the bound conformation of the peptide with G-quadruplex structure (Fig. 2a). Qualitatively, as the conformation of the G-quadruplex molecules is induced to different degrees by the binding of the QW10 peptide hence, the CD spectrum of the complex between the G4 and the peptide at particular molar ratio was subtracted from the reference containing the same peptide molar ratio. We observed a slight decrement of CD intensity at 260 nm upon the titration of QW10. However, these changes were very small and the overall CD spectra was almost similar. These results indicated that QW10 was not binding to the c-Myc promoter G4 significantly in the presence of Na^+^. These results are consistent with our recently published results where we have shown that the Human telomere G4 was not binding with QW10 in the presence of Na^+^.^31^ In contrast, c-Myc promoter G4 in the presence of K^+^ exhibited a strong positive peak around 268 nm and negative peak at 239 nm indicating the same characteristics of parallel G-quadruplex^27^ (Fig. 2b). On titrating this G4 formed in the presence of K^+^ with an increasing concentration of QW10, positive peak at 268 nm shifted towards 255 nm with subsequent increase in CD intensity at this shifted wavelength and negative peak at 240 nm completely disappeared. We also observed two isodichoric points, one was at 294 nm and other was at 258 nm. This indicated that peptide was binding strongly to K^+^ formed c-Myc G4 and changing its conformation. As CD spectra of control G-rich sequence forms parallel intramolecular G-quadruplex so it is quite possible that the peptide binding might have induced the significant blue shift upon binding and this new structure may be due to the new topology of G-quadruplex–peptide complex which has to be explored in future. As major changes in c-Myc promoter G4 were observed with K^+^, hence we have extended these studies with K^+^ under cell mimicking molecular crowding conditions.

#### 2.7.3 Structural changes in human c-Myc promoter G4 in the presence of K^+^ with and without peptide under cell mimicking molecular crowding conditions

CD spectra of c-Myc G4 was recorded in the presence of K^+^ under cell-mimicking molecular crowding conditions using 40 wt% PEG 200 (Fig. 2c) and 20 wt% PEG 8000 (Fig. 2d). CD spectrum is characterized by a positive peak at 266 nm and negative peak at 238 nm typically observed for a parallel G-quadruplex in the presence of 100 mM K^+^ and 40 wt% PEG 200.^29^ These CD signatures are consistent with the previously published crystal structure of the same sequence adopts a fully parallel arrangement with potassium cations occupying the central channel.^29,30^ Next, we have titrated c-Myc G4 with increasing concentrations of QW10 (Fig. 2a). We observed a slight decrement of CD intensity in the positive peak at 266 nm in control and interestingly this positive peak gradually shifted towards 254 nm, 250 nm and 244 nm and negative peak at 238 nm shifted to 231 nm on subsequent addition of peptide. In addition, we observed prominent isodichoric point at 260 nm. These results indicate that QW10 was bound to the c-Myc G4 and significantly altered its structure. We have also extended the interaction study with c-Myc G4 in the presence of 100 mM K^+^ and 20 wt% PEG 8000. CD spectrum was characterized by a positive peak at 267 nm and negative peak at 239 nm typically observed for parallel G-quadruplex.^26^ On titrating with QW10 positive peak at 267 nm gradually shifted towards 269 nm on small increment of peptide and shifted further towards 265 nm, 243 nm, 242 nm and finally to 246 nm. The negative peak at 239 nm gradually keeps on decreasing and disappeared finally. Interestingly, we observed two prominent isodichoric points one is at 261 nm and another is at 282 nm. These CD results confirmed that c-Myc G4 adopted a parallel G-quadruplex structure in the absence of peptide and adopted new parallel topology due to the formation of parallel G-quadruplex–peptide complex irrespective of the nature of cosolutes. This possibility will be further explored by UV thermal melting and native gel electrophoresis data in the following sections.

#### 2.7.4 Thermodynamic analysis of the human c-Myc promoter G4 structure with and without peptide under dilute and cell mimicking molecular crowding condition

**Fig. 3:**
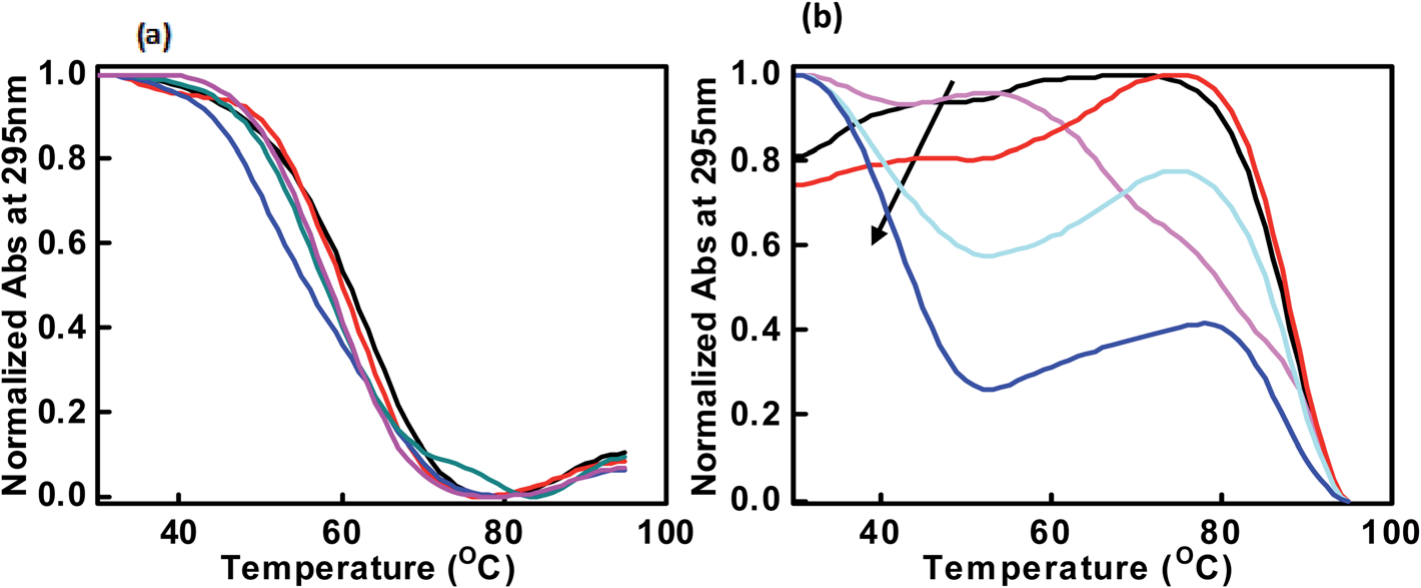
Normalized UV melting curves of 4 μM c-Myc promoter G4 in buffer containing NaCl (100 mM) (a) and 100 mM KCl (b), 0.5 mM EDTA without any additive (black line), c-Myc promoter G4 : QW10 ratio (1 : 1) (redline), (1 : 2) (pink line), (1 : 5) (green line) and (1 : 10) (blue line).

Next, we explored the thermal stability of the DNA structures with and without peptide. Fig. 3a shows normalized UV melting profile of 4 μM c-Myc promoter G4 in the buffer containing 100 mM NaCl or KCl in the absence and presence of QW10. The ratios of c-Myc promoter G4 are (1 : 0, 1 : 1, 1 : 2, 1 : 5 and 1 : 10) respectively (Fig. 3a). The melting temperature (*T*_m_) was evaluated by a curve fitting procedure as described previously.^27^ The melting curves with a single transition were obtained in all conditions. The *T*_m_ of the c-Myc promoter G4 was decreased initially from 63.1 °C, 61.1 °C, 51.1 °C, with the DNA peptide ratio of (1 : 0, 1 : 1, 1 : 2) and increased further to 57.1 °C, 58.1 °C at (1 : 5 and 1 : 10) respectively. These results indicated that peptide did not bind significantly with c-Myc promoter G4 structure in the presence of Na^+^. These results are consistent with our CD results showing the signatures of parallel G-quadruplex in control with slight decrease in CD intensity on increasing the peptide concentration in the presence of Na^+^ as shown above (Fig. 2a).

On the contrary, we obtained a well resolved biphasic melting curve in the buffer containing KCl in the absence and presence of QW10. We proposed that the lower transition (*T*_m1_) at 48.0 °C is due to the melting of biomolecular structure while the upper transition (*T*_m2_) at 88.1 °C is due to the melting of unimolecular G-quadruplex structure. Next, melting profiles were recorded with DNA: peptide ratio as (1 : 1, 1 : 2, 1 : 5, 1 : 10 and 1 : 20). The *T*_m_ of lower transition (*T*_m1_) decreased gradually from 48.1 °C, 48.0 °C, 42.0 °C, 39.1 °C and 37.1 °C while the *T*_m_ of upper transition (*T*_m2_) was increased from 87.1 °C, 81.1 °C, 88.1 °C, 88.7 °C and 90.1 °C respectively (Fig. 3b). Therefore, the decrease in *T*_m1_ value is due to the melting of unimolecular G-quadruplex structure which is distinct from G-quadruplex–peptide complex and increase in *T*_m_ of upper transition is due to G-quadruplex–peptide complex. This might be possible that peptide binding unwinded the G-quadruplex initially and again reformed the G-quadruplex–peptide complex with new topology. This possibility will be further explored by native gel electrophoresis data in the following section. Next, we have recorded the thermal melting profile of c-Myc promoter G4 in the presence of K^+^ and cell mimicking molecular crowding conditions. The *T*_m_ of the control with DNA : peptide ratio as (1 : 0) was 91.6 °C and 91.8 °C in the presence of 100 mM K^+^ and 40 wt% PEG 200 or 20 wt% PEG 8000 (Fig. S1a and b†) respectively. There was relatively 3 °C and 1 °C change with (DNA : peptide ratio of 1 : 1) under both molecular crowding conditions. Interestingly, c-Myc promoter G4 was completely adopted new conformation on further increasing the QW10 concentration and it was not possible to evaluate the *T*_m_ values (Fig. S1a and b†). These *T*_m_ results were in good agreement with those obtained by CD measurements (Fig. 2c and d).

#### 2.7.5 Native gel electrophoresis of the c-Myc promoter G4 with peptide under dilute and cell mimicking molecular crowding conditions

**Fig. 4:**
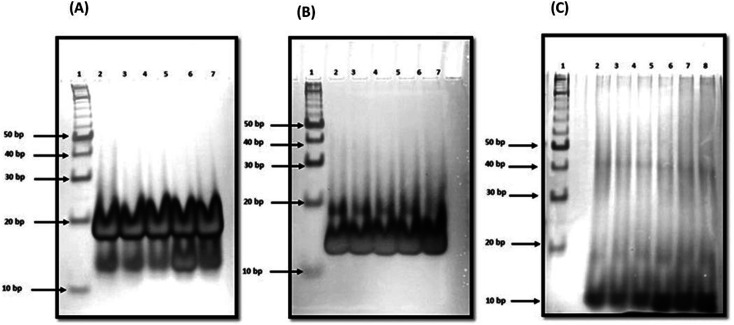
Native gel electrophoresis of 15 μM c-Myc G4 in 30 mM sodium cacodylate buffer (pH 7.0), 1 mM EDTA, 100 mM NaCl (a) 100 mM KCl and 40 wt% PEG 200 (b), 100 mM KCl and 20 wt% PEG 200 (c). Lane 1, 10 bp ladder, lane 2 DNA : peptide (1 : 0), lane 3 DNA :  peptide (1 : 1), lane 4 DNA : peptide (1 : 2), lane 5 DNA : peptide (1 : 5) respectively. Lane 6 DNA : peptide (1 : 10) respectively.

In order to reveal a molecular mechanism of the c-Myc promoter G4 and QW10 binding, we further investigated the complex in the presence of Na^+^ using non-denaturating PAGE (Fig. 4). The PAGE experiment can discriminate molecularity of c-Myc promoter G4 and c-Myc promoter G4–QW10 complex. The electrophoretogram in Fig. 4 shows the structural status of c-Myc promoter G4 in the presence and absence of QW10. 10 bp DNA ladder was used to compare their electrophoretic mobility. The lane 2 of Fig. 4a displayed two bands, major band equivalent to 10 or 20 base pairs indicated that c-Myc promoter G4 folds into dimeric structure and the minor band equivalent to 10 bp corresponded to unimolecular G-quadruplex structure. Next, we have checked the migration of c-Myc promoter G4 folding in the presence of QW10. The c-Myc promoter G :  QW10 were in the ratio of (1 : 1) (lane 3), (1 : 2) (lane 4), (1 : 5) (lane 5), and (1 : 10) (lane 6) respectively. We observed two bands between 10 to 20 bp on comparing with 10 bp band in all four lanes (lane 3 to lane 6). There was no change in band intensity of upper band while band intensity of lower band increased in lane 5 and lane 6 on increasing the peptide concentration. These gel results suggested that peptide is binding with unimolecular structure in the presence of Na^+^. Next, we have checked the structural state of c-Myc promoter G4 in the presence of K^+^ with and without QW10. The lane 2 of Fig. 4b displayed again the two bands which migrated equivalent to 10 or 20 base pairs. Here, the major band corresponded to unimolecular G-quadruplex structure and minor band to bimolecular structure. On increasing the peptide concentration, there was continuous decrease in band intensity of minor upper band in lane 3 to lane 5 and this band completely disappeared in lane 6. In addition, the band intensity of the lower band increased again which showed the preference of peptide binding to unimolecular structure. These results are consistent with our CD results obtained on titrating the c-Myc G4 with an increasing concentration of QW10. Presence of major blue shift of 20 nm in positive peak and isodichoric point at 260 nm confirmed the binding of peptide with parallel G 4 structure (Fig. 2b). We have also checked the structural state of c-Myc promoter G4 in the presence of K^+^ and 40 wt% PEG 200 with and without QW10 (Fig. 4c). Three bands were observed, out of which two bands migrated equivalent to 10 to 20 bp while the third band migrated equivalent to 40 bp. The parallel topology was shown to be favoured in solutions containing molecular crowding conditions. On increasing the peptide concentration from lane 3 to lane 6, there was no change in band intensity of upper band while the band intensity of middle and lower band increased. This observation leads to the possibility of the preference of peptide binding with bimolecular and unimolecular topology under molecular crowding conditions. We have also performed one control experiment to see the changes in W.C bonded DNA duplex and C-tetraplex (i-motif) upon QW10 binding in 100 mM KCl, (pH 7.0 and pH 5.7) respectively (Fig. S2†). Duplex was prepared by mixing human telomere G-rich and C-rich strand in (1 : 1) ratio at (pH 7.0) (lane 2) and C-tetraplex was prepared by taking human telomeric C-rich strand at pH (5.7) (lane 4) alone or with QW10 in DNA : peptide (1 : 10) ratio (lane 3 and lane 5) respectively. We observed one band migrated between 20 and 30 bp (lane 2) corresponded to the formation of DNA duplex. Interestingly, after addition of peptide in 1 : 10 ratio, we observed two bands, upper band between 20 and 30 bp and lower band at 10 bp which clearly indicated that W.C duplex was destabilized upon peptide binding. We observed single band migrated between 10 bp and 20 bp in lane 4 (pyrimidine rich DNA) and lane 5 pyrimidine rich DNA : peptide (1 : 10) ratio. These results indicated that human telomeric c-rich strand folded into bimolecular i-motif structure and there was no change in mobility upon peptide addition which confirmed that peptide is not binding to C-tetraplex.

#### 2.7.6 Exploration of peptide binding to c-Myc promoter G4 DNA by fluorescence measurements

**Fig. 5:**
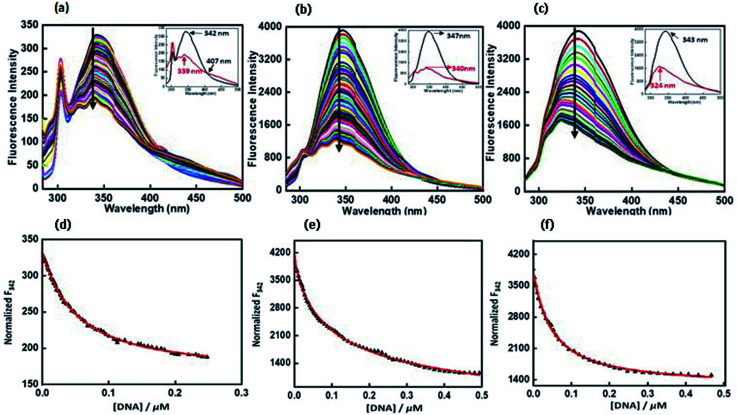
Emission spectra of QW10 peptide with c-Myc promoter G4 in buffer (pH 7.0) containing, 0.5 mM EDTA, 100 mM KCl (a) 100 mM KCl and 40 wt% PEG 200 (b) 100 mM KCl and 20 wt% PEG 8000 at 25 °C. QW10 = 4 μM was titrated with equimolar preformed c-Myc G4 structure in an increasing concentration. Normalized fluorescence intensity at 342 nm (*F*_342_), 347 nm (*F*_347_), 343 nm (*F*_343_) with various concentrations of c-Myc G4 in KCl (d), KCl and 40 wt% PEG 200 (e), KCl and 20 wt% PEG 8000 (f) at 25 °C respectively.

Fluorescence titration experiments were employed to study the binding affinities of QW10 with c-Myc promoter G4 DNA. The fluorescence emission of QW10 was examined at the emission maximum in their unbound form. Upon excitation at 275 nm, the peptide produced an emission band due to the presence of tryptophan residue with a maxima centred at 342 nm and minima at 319 nm (Fig. 5a). The c-Myc promoter G4 prepared in the presence of potassium cations was added to the peptide until very small changes in fluorescence spectra were observed. On addition of DNA, the observed changes in the fluorescence intensity depicted the binding of QW10 to c-Myc promoter G4 and generated G-quadruplex–peptide complex. We observed that the fluorescence of QW10 was quenched with maxima peak shifted to 339 nm and minima peak shifted to 319 nm on increasing the DNA concentration indicating that the tryptophan was intercalating within G-quadruplex planes during binding. The value of binding constant (*K*_b_) was evaluated as 0.05 ± 0.2 μM (Fig. 5d). Furthermore, to understand the specificity and the binding affinity of QW10 with c-Myc G4 promoter, we have also performed the fluorescence titration experiment in buffer containing 100 mM K^+^ and 40 wt% PEG 200 (Fig. 5b) or 20 wt% PEG 8000 (Fig. 5c). There was significant decrease in fluorescence intensity of peptide along with 7 nm blue shift and 21 nm blue shift in CD on successive addition of c-Myc G4 with K^+^ and 40 wt% PEG 200 (Fig. 2c) and 20 wt% PEG 8000 (Fig. 2d) respectively. These results further confirmed the formation of a G4–peptide complex under molecular crowding conditions. The (*K*_b_) values calculated were 0.12 ± 0.1 μM and 0.05 ± 0.3 μM with K^+^ and 40 wt% PEG 200 (Fig. 5e). and 20 wt% PEG 8000 (Fig. 5f) respectively. These *K*_b_ values confirmed the strong affinity of the peptide with c-Myc promoter G4. This observed difference in the affinity of QW10 with c-Myc G4 under low and high molecular weight co-solute might be due to the differences in water activity.

To check the effect of QW10 peptide on Watson–Crick base-pairing as well as on i-motif structure, we have also prepared the Watson–Crick base paired duplex (5′-GGGTTAGGGTTAGGGTTAGGGTTA-3′) purine strand and (5′-TAACCCTAACCCTAACCCTAACCC-3′) by mixing purine and pyrimidine rich strand in 1 : 1 ratio at neutral pH (Fig. S3a†) and i-motif structure using pyrimidine rich single strand (5′-TAACCCTAACCCTAACCCTAACCC-3′) at acidic pH (5.7). We have also recorded its fluorescence with and without peptide (Fig. S3c†). There was decrease in fluorescence intensity with *K*_b_ value as 1.30 ± 0.7 μM (Fig. S3b†) and 17.74 ± 0.8 μM (Fig. S3d†) respectively. So, overall fluorescence results indicated the preference of peptide binding with Hoogsteen bonded G-quadruplex structure rather than Watson–Crick hydrogen bonded and i-motif structure.

#### 2.7.7 QW10 peptide inhibited cell proliferation in human adenocarcinoma breast cancer cells (MDA-MB-231)

**Fig. 6:**
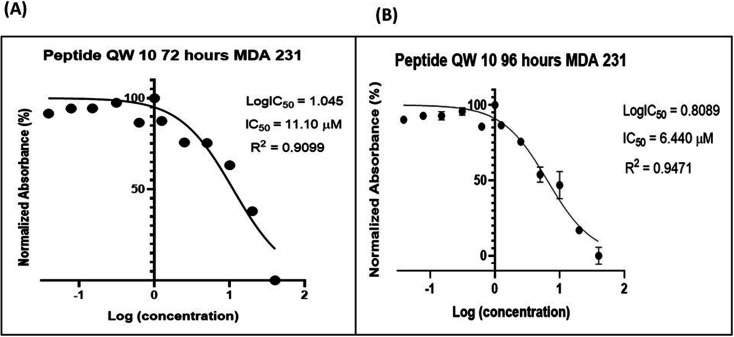
QW10 demonstrated the anti-proliferative effects on human breast adenocarcinoma cells MDA-MB-231. Difference in absorbance is due to the different rate of formazan formation at two time points 72 hours (A) and 96 hours (B) of cells incubated with QW10 at concentrations ranging from 40 μM down to 39 nM. The data is analysed by comparing the changes in the absorbance from cells with media only (control).

The interesting biophysical observations prompted us to perform cellular studies to extrapolate the findings of QW10 induced structural transition, stabilization and modulation of molecular recognition properties of c-Myc quadruplex structure to c-Myc transcription. We have evaluated whether QW10 exhibit anti-proliferative effect against highly proliferative human cancer cell line MDA-MB-231 cells (Fig. 6) and also compared with HEK-1 cells (Fig. S4†). To elucidate the said effect of QW10 peptide, various time points and a range of concentration of the peptide were screened to determine the effective time points and peptide concentration(s) with maximum anti-proliferative efficacy of the peptide demonstrating less than 50% cell viability (IC_50_). The results showed that QW10 significantly inhibited the proliferation (affecting cell viability) of human adenocarcinoma cells in a dose-dependent manner with IC_50_ values of 11.10 μM and 6.44 μM after 72 (Fig. 6a) and 96 hours (Fig. 6b) respectively. Moreover, to determine whether QW10 peptide impacts cellular toxicity in normal cells or not, we have checked the proliferation in HEK-1 cells with various concentration of the peptide and IC_50_ observed was 96.05 μM after 72 hours of incubation shown in (Fig. S4†). These results suggested that the QW10 peptide inhibited cell proliferation in this human adenocarcinoma cells and found to be non-toxic to human embryonic kidney cells.

#### 2.7.8 Effect of QW10 peptide on c-Myc gene expression in MDA-MB-231 using quantitative real-time PCR

**Fig. 7:**
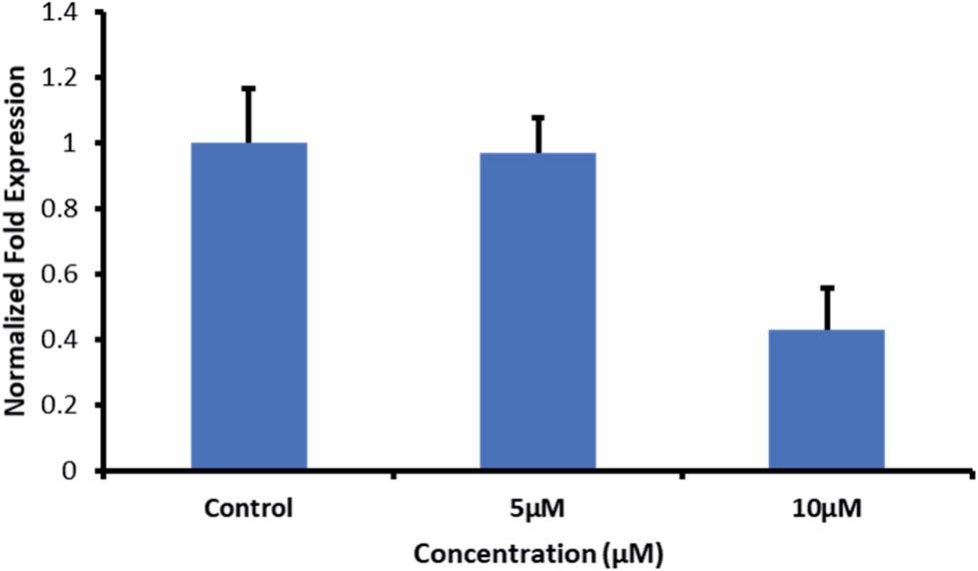
Effect of QW10 on c-Myc mRNA levels in MDA-MB231 cells. Fold change in the c-Myc transcripts assessed through real time PCR after 24 h of treatment of QW10 (5 μM and 10 μM) MDA-MB 231 cells. The fold change represents the log2-fold change of c-Myc transcripts with respect to internal reference gene for treated samples *versus* control sample, which received no QW10 treatment. Cells were exposed to 5 μM and 10 μM for 24 hours. Non-treated cells were maintained at the same conditions to compare the gene expression profile. qPCR was performed using a final volume reaction of 10 μL containing forward and reverse primer. 1× SYBR Green master mix, 40 cycles at 95 °C for 10 s, 64 °C for 30 s and 72 °C for 15 s. Melting curve analysis was performed at ramping from 60 °C to 90 °C and rising by 5 °C every 2 s. Gene expression variations were analysed in terms of fold induction concerning the untreated control cells by 2^ΔΔCT^ method. The data represent the mean values ± SDs from three separate experiments.

The real-time polymerase chain reaction is an essential tool to understand the gene expression profile of cancer cells to predict the clinical outcome, to find out the biological alterations and to propose the personalized targeted therapies to patients. We applied the qPCR array to analyze the response of MDA-MB-231 cells towards QW10 peptides. Most of the genes repressed by c-Myc are involved in cell proliferation, senescence, apoptosis and cell cycle arrest.^32^ As the c-Myc G4 sequence was located in the promoter region and formed parallel G-quadruplex, therefore, the its binding with QW10 could increase the stability of the G-quadruplex structure. This may result in the downregulation of oncogene c-Myc transcription, which would be identified using real-time RT-PCR. To test the above hypothesis and to understand the effect of QW10 peptide on c-Myc gene expression, MDA-MB-231 were tested (Fig. 7). After treatment of c-Myc G4 with QW10 at two different concentrations (5 μM or 10 μM) for 24 h, the total RNA was extracted and reverse transcripted to cDNA. This cDNA was then used as a template for specific RCR amplification of the c-Myc gene. As shown in Fig. 7 the binding of QW10 with c-Myc promoter G4 down regulated its expression by 2.5 fold which may be due to the ability of QW10 to bind and stabilize the c-Myc G4 promoter.

## 3 Conclusion

We report the interaction of designed peptide as the basis of a novel class of c-Myc G4 binder that exhibits highly specific potential for stabilizing the potassium form of c-Myc G4 over the analogous sodium form. The proposed mechanisms for the binding of the QW10 peptide to c-Myc G4 was the hydrogen bonding with the G-base of G-quadruplex and the side chain of the glutamine followed by the intercalation of tryptophan in G-quartet plane. We observed the significant changes in CD spectra on titrating the c-Myc G4 with QW10 peptide with 20 nm blue shift along with two distinct isodichoric points. These CD changes in molar ellipticity clearly indicate that peptide is binding to the c-Myc G4. Changes in molar ellipticity were observed in the presence of both monovalent ions used during studies in sodium or potassium formed G-quadruplex indicating that the binding of peptide is conformation specific. As the structure of c-Myc G4 is different in K^+^ and Na^+^, therefore, peptide recognizes and binds to these structures differently. We observed destabilization of dimeric structure and stabilization to unimolecular G-quadruplex structure on peptide binding in the presence of K^+^ in comparison to Na^+^. This is further confirmed by the presence of higher molecular weight G-quadruplex–peptide complexes observed in Native PAGE data. Fluorescence data also supported the binding of QW10 peptide with c-Myc G4 with *K*_b_ values 0.05 ± 0.2 μM, 0.12 ± 0.1 μM and 0.05 ± 0.3 μM in the presence of K^+^ alone or with 40 wt% PEG 200 or 20 wt% PEG 8000. as discussed above. The cellular cytotoxicity results of QW10 peptide against breast cancer cell lines was also evaluated and IC_50_ values were 11.10 μM and 6.44 μM after 72 and 96 hours respectively. Further, we found that the binding of QW10 with c-Myc promoter G4 could down-regulate the transcription of oncogene c-Myc by 2.5 fold in human breast cancer cell lines. Based on CD, UV-thermal melting, native PAGE and fluorescence and cellular studies, we conclude that the peptide has potential application in the exploration of anticancer drugs for the recognition of c-Myc G4 and offers a new approach for anti-cancer therapy by G-quadruplex mediated inhibition of c-Myc expression. Work to investigate the exact structure of new topology of c-Myc promoter G4peptide complex and its thermodynamics is under progress in our laboratory.

## Funding

We thank “Department of Biotechnology (DBT)”, Govt of India for research funding to this project (SAN No. 102/IFD/SAN/864/2018-2019).

## Conflicts of interest

The authors declare no competing financial interest.

## Supplementary Material

RA-012-D2RA00535B-s001
