# Supplementary material for: Significant structural change in human c-Myc promoter G-quadruplex upon peptide binding in potassium
Source: RSC Adv. 2022 Mar 8;12(13):7594–604. doi: 10.1039/d2ra00535b (PMC8982240; doi:10.1039/d2ra00535b)
Supplement: RA-012-D2RA00535B-s001 [file RA-012-D2RA00535B-s001.pdf]

Electronic Supplementary Material (ESI) for RSC Advances.
This journal is © The Royal Society of Chemistry 2022

Significant structural change in human c-Myc promoter G-quadruplex upon peptide binding in potassium

Nikita Kundu[a], Taniya Sharma[a], Sarvpreet Kaur[a], Mamta Singh[b,] Vinit Kumar[b], Uttam Sharma[c], Aklank Jain[c] Jadala Shankaraswamy[d], Daisuke Miyoshi[e], Sarika Saxena[a*]

[a]Amity Institute of Biotechnology, Amity University Uttar Pradesh, Noida 201313, India.

[b]Amity Institute of Molecular Medicine and Stem Cell Research, Amity University Uttar Pradesh, Noida 201313, India.

[c]Department of Animal Sciences, Central University of Punjab, Bathinda.

[d]Department of Fruit Science, College of Horticulture, Mojerla, Sri Konda Laxman Telangana State Horticultural University, 509382, Telangana, India.

[c]Faculty of Frontiers of Innovative Research in Science and Technology (FIRST), Konan University, 7-1-20 Minatojima-minamimachi, Chuo-ku, Kobe, Hyogo 650-0047, Japan.

Note: Nikita Kundu, Taniya Sharma, and Sarvpreet Kaur share equal contribution.

Address of Correspondence: Structural Biology Lab, Sector-125, Expressway Highway, Amity Institute of Biotechnology, Amity University Uttar Pradesh, Noida 201313, India. Phone Number: 0120-4735600,

Email: ssaxena1@amity.edu; sarikaigib@yahoo.co.in.

Supporting Information

Table of Contents 2

1. Figure S1 Normalized UV melting curves of 4 μM c-Myc promoter G4 in buffer containing 100 mM KCl with 40 wt% PEG 200 and 100 mM KCl with 20 wt% PEG 8000**.** **3**

2. Figure S2 Native Gel Electrophoresis of 4μM HTPu.HTPy (Duplex) in 30 mM sodium cacodylate buffer (pH 7.0) and HTPy (pH 5.7) with and without peptide **4**

3. Figure S3: Emission spectra of QW10 peptide with HTPu.HTPy duplex in buffer (pH 7.0) (a) and HTPy in buffer (pH 5.7) with and without peptide **5**

4. Figure S4. QW10 demonstrated the normal effects on human embryonic kidney cells HEK-2 (A). **6**

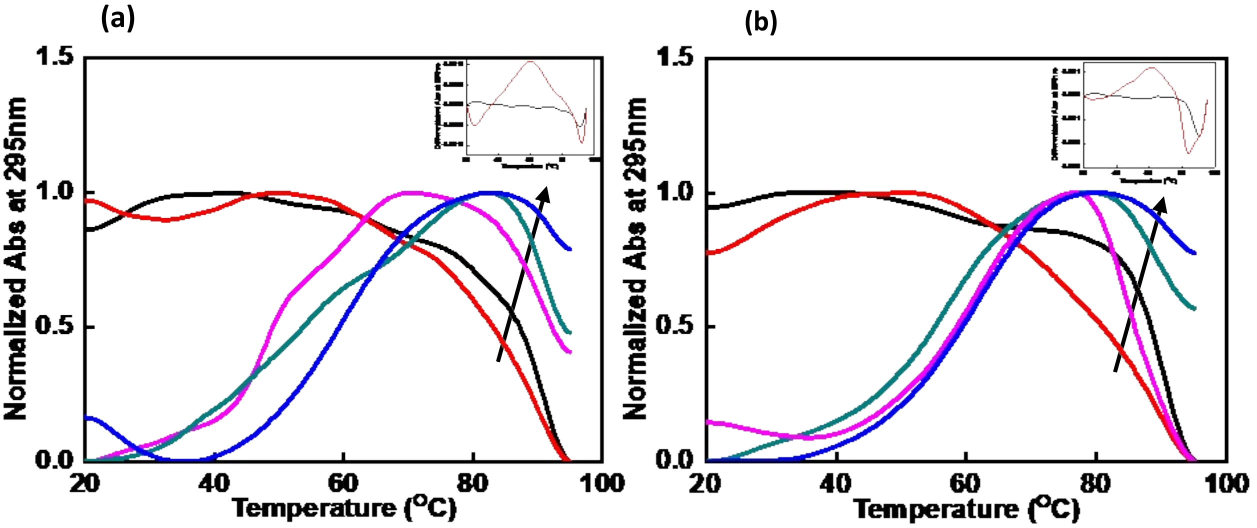

Figure S1 Normalized UV melting curves of 4 μM c-Myc promoter G4 in buffer containing 100 mM KCl with 40 wt% PEG 200 (a) and 100 mM KCl with 20 wt% PEG 8000 (b), 0.5 mM EDTA without any additive (black line), c-Myc promoter G4:QW10 ratio (1:1) (red line), (1:2) (pink line), (1:5) (green line) and (1:10) (blue line).

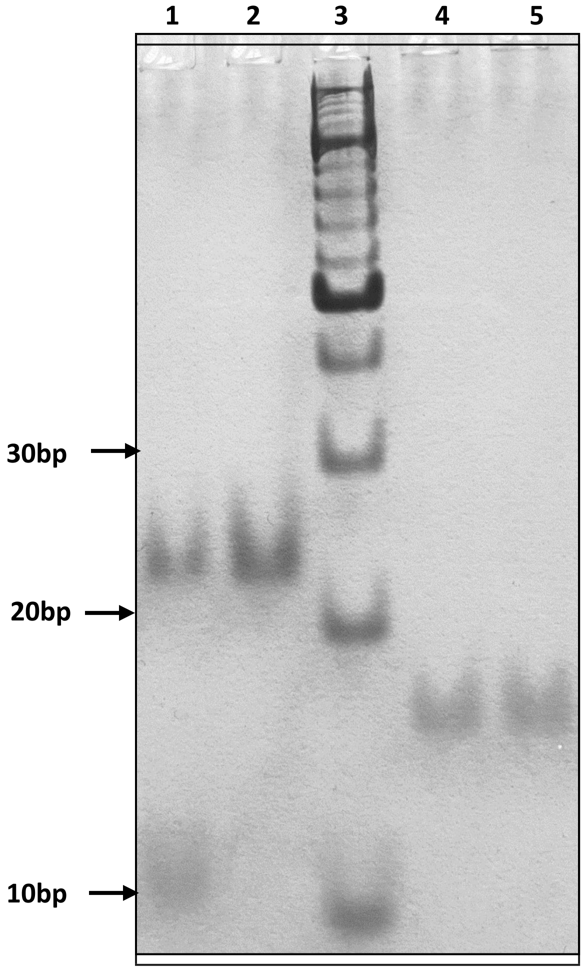

Figure S2: Native Gel Electrophoresis of 4μM HTPu.HTPy (Duplex) in 30 mM sodium cacodylate buffer (pH 7.0) and HTPy (pH 5.7), 1 mM EDTA, 100 mM KCl. Lane 1 is DNA duplex: peptide (1:10), Lane 2 is DNA duplex, Lane 3 is 10 bp ladder, Lane 4 is HTPy: Peptide (1:10) and Lane 5 is HTPy only.

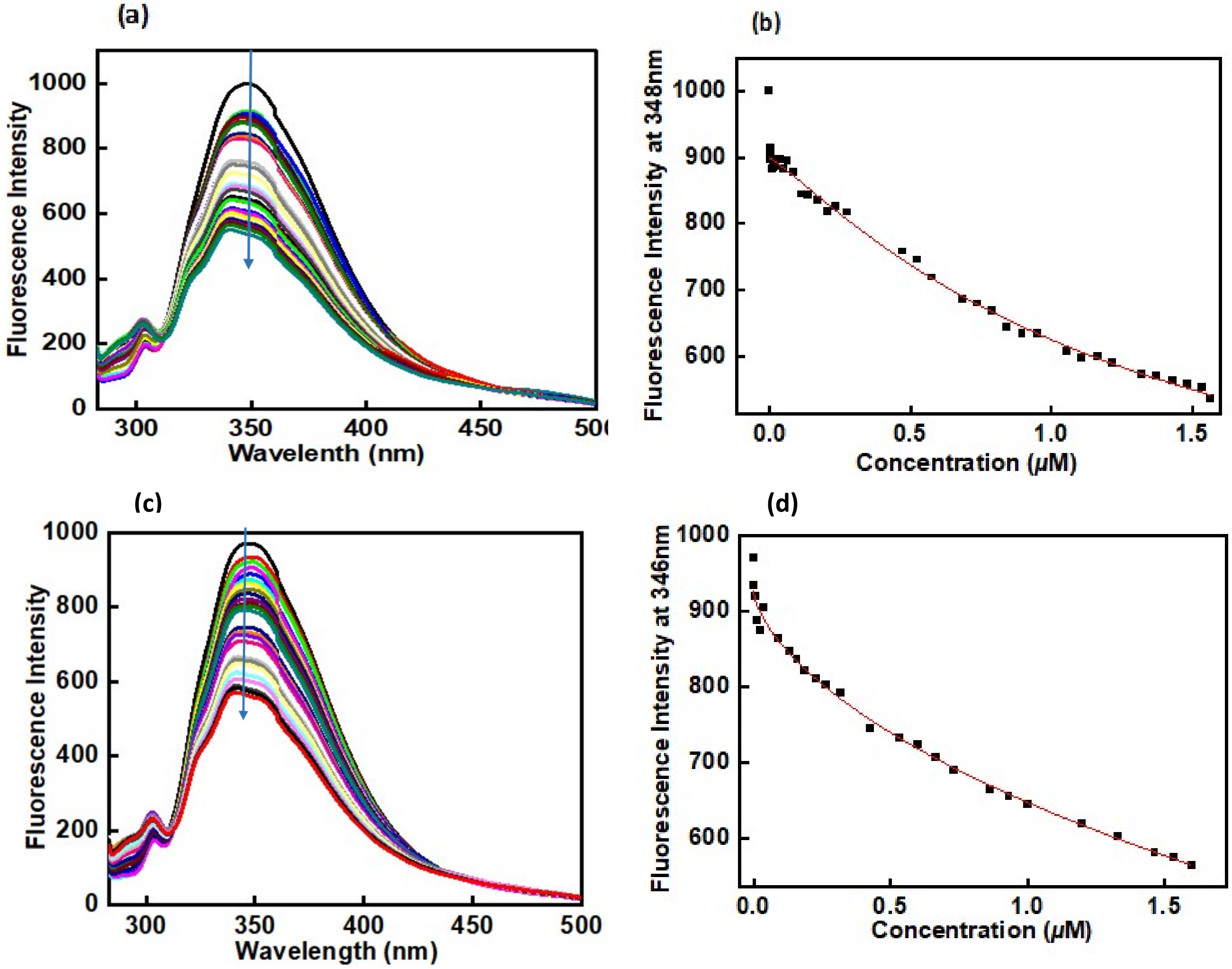

Figure S3: Emission spectra of QW10 peptide with HTPu.HTPy duplex in buffer (pH 7.0) (a) and HTPy in buffer (pH 5.7) (c) containing 0.5 mM EDTA, 100 mM KCl at 25°C. QW10 = 4μM was titrated with equimolar preformed HTPu.HTPy duplex and i-motif structure in an increasing concentration. Normalized fluorescence intensity at 348 nm (F_{348}), 346 nm (F_{346}), with various concentrations of HTPu.HTPy duplex in KCl (b), HTPy i-motif (d) at 25°C respectively.

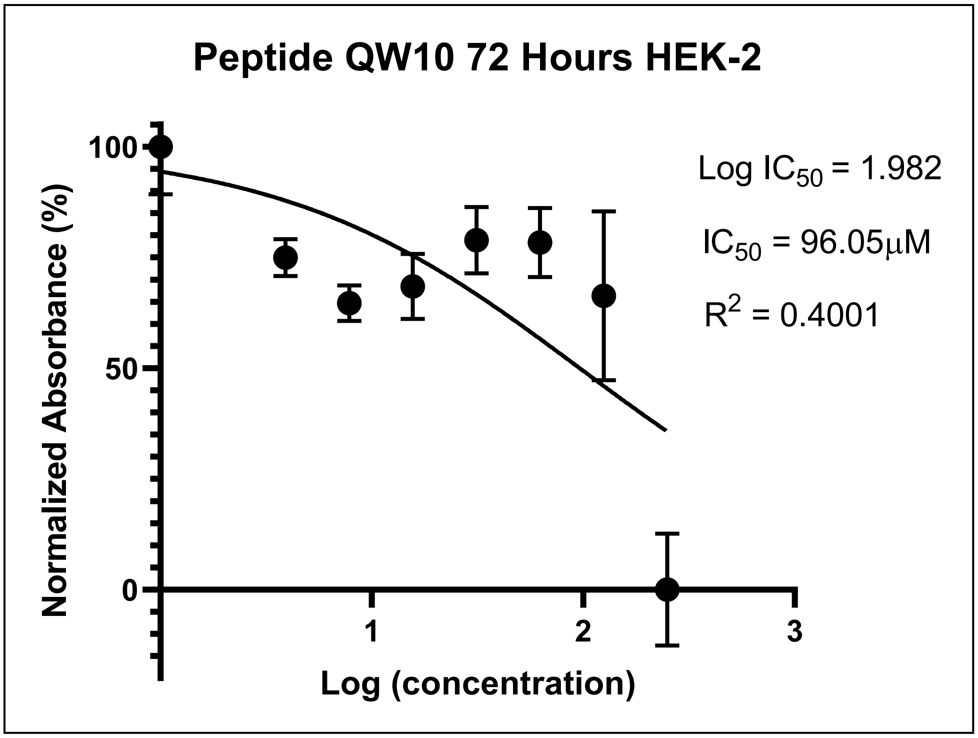

Figure S4. QW10 demonstrated the normal effects on human embryonic kidney cells HEK-2 (A). Difference in absorbance is due to the different rate of formazan formation at 72 h of cells incubated with QW10 at concentrations ranging from 40μM down to 39 nM. The data is analyzed by comparing the changes in the absorbance from cells with media only (control).
